# Intact salicylic acid signalling is required for potato defence against the necrotrophic fungus *Alternaria solani*

**DOI:** 10.1007/s11103-020-01019-6

**Published:** 2020-06-19

**Authors:** Sophie M. Brouwer, Firuz Odilbekov, Dharani Dhar Burra, Marit Lenman, Pete E. Hedley, Laura Grenville-Briggs, Erik Alexandersson, Erland Liljeroth, Erik Andreasson

**Affiliations:** 1 Department of Plant Protection Biology, Swedish Agricultural University, Alnarp, Sweden; 2 Department of Cell and Molecular Sciences, Genome Technology, James Hutton Institute, Dundee, Scotland UK

**Keywords:** Early blight, *Alternaria solani*, SA, JA, NahG, Coi1, Potato, *Solanum tuberosum*

## Abstract

**Key Message:**

Using disease bioassays and transcriptomic analysis we show that intact SA-signalling is required for potato defences against the necrotrophic fungal pathogen *Alternaria solani*.

**Abstract:**

Early blight, caused by the necrotrophic fungus *Alternaria solani,* is an increasing problem in potato cultivation. Studies of the molecular components defining defence responses to *A. solani* in potato are limited. Here, we investigate plant defence signalling with a focus on salicylic acid (SA) and jasmonic acid (JA) pathways in response to *A. solani*. Our bioassays revealed that SA is necessary to restrict pathogen growth and early blight symptom development in both potato foliage and tubers. This result is in contrast to the documented minimal role of SA in resistance of *Arabidopsis thaliana* against necrotrophic pathogens. We also present transcriptomic analysis with 36 arrays of *A. solani* inoculated SA-deficient, JA-insensitive, and wild type plant lines. A greater number of genes are differentially expressed in the SA-deficient mutant plant line compared to the wild type and JA- insensitive line. In wild type plants, genes encoding metal ion transporters, such as copper, iron and zinc transporters were upregulated and transferase-encoding genes, for example UDP-glucoronosyltransferase and Serine-glyoxylate transferase, were downregulated. The SA-deficient plants show upregulation of genes enriched in GO terms related to oxidoreductase activity, respiratory chain and other mitochondrial-related processes. *Pathogenesis-related* genes, such as genes encoding chitinases and PR1, are upregulated in both the SA-deficient and wild type plants, but not in the JA-insensitive mutants. The combination of our bioassays and the transcriptomic analysis indicate that intact SA signalling, and not JA signalling, is required for potato defences against the necrotrophic pathogen *A. solani*.

**Electronic supplementary material:**

The online version of this article (10.1007/s11103-020-01019-6) contains supplementary material, which is available to authorized users.

## Introduction

*Alternaria solani* is a necrotrophic pathogen that causes early blight in tomato and potato. In potato, *A. solani* can infect the leaves resulting in poor tuber yield, but it can also infect the tubers (Sherf and MacNab 1986; Rotem 1994; Thomma 2003). Studies have estimated that if the disease in the field is left uncontrolled, yield losses can reach up to 50% (Leiminger and Hausladen 2012). A recent study showed that early blight is one of only four pests and pathogens affecting potato production that causes global crop losses higher than one percent (2.6%; Savary et al. 2019). Currently, early blight is controlled by the application of fungicides, even though fungicidal resistance in *A. solani* populations has been reported in several countries (Rosenzweig et al. 2008; Leiminger et al. 2014; Odilbekov et al. 2016, 2019). Additionally, a lack of identified resistance genes combined with a corresponding lack of resistant varieties, together with limited knowledge of the mechanisms of defence against *A. solani*, contribute to a severe disease problem.

In response to pathogens, plants protect themselves by both constitutive barriers, such as the cell wall, and inducible defence systems, such as the release of reactive oxygen species. Induced defences involve activation of complex signalling cascades involving phytohormones, such as jasmonic acid (JA) or salicylic acid (SA). These complex signalling cascades and their cross-talk play important roles in plant defence responses, depending on the lifestyle of the pathogen. In the model plant *Arabidopsis thaliana,* studies suggest that the SA signalling pathway is essential for resistance to biotrophic and hemibiotrophic pathogens, and that the JA pathway is efficient against necrotrophs and biting insects (McDowell and Dangl 2000). The critical role for SA in resistance to biotrophic pathogens was shown in studies using *A. thaliana* mutants that fail to produce SA. Screening of *NahG* transgenic lines that can’t accumulate SA and *Arabidopsis* mutant plants impaired in SA signalling, such as *enhanced disease susceptibility1* (*eds1*) and *SA induction deficient 2* (*sid2*), showed enhanced susceptibility against different biotrophic pathogen infections. (Kunkel and Brooks 2002; Glazebrook 2005). In contrast, *A. thaliana* JA signalling mutants, e.g. *coronatine insensitive 1* (*coi1*) and *jasmonic acid resistant 1* (*jar1*) show enhanced susceptibility to necrotrophic pathogens (Thomma et al. 1998; Kunkel and Brooks 2002). Multiple studies determined the primary mode of interaction between these pathways to be antagonistic (Reymond and Farmer 1998; Pieterse and van Loon 1999; Kunkel and Brooks 2002; Rojo et al. 2003). Niki et al. (1998) showed that the exogenous application of methyl jasmonate (MeJA) on tobacco leaves results in inhibition of SA-induced *pathogenesis-related* (PR) gene expression. Additionally, it was shown that SA treatment of tomato plants reduced JA-induced proteinase inhibitor gene expression (Doares et al. 1995). However, there is substantial evidence that the SA and JA signalling pathways form a complex network of positive and negative interactions (Robert-Seilaniantz et al. 2011; Yang et al. 2015; Vos et al. 2013, 2015; Koo et al. 2020). Several studies provided evidence of synergistic interactions and cross-talk between SA and JA pathways (Schenk et al. 2000; van Wees et al. 2000; Beckers and Spoel 2006; Mur et al. 2006). Notably, potato leaves have been shown to contain high basal levels of salicylic acid that are about 100-fold higher than *A. thaliana* and approximately 15 to 20-fold higher than that of *Solanum lycopersicum* leaves. Even though potato tubers do not contain as high levels of total SA as the potato leaves, the levels are still 2–4-fold higher than the levels found in tomato leaves (Yu et al. 1997; Navarre and Mayo 2004; López-Gresa et al. 2016), indicating species- and tissue-specific differences in SA signalling. Additionally, SA perception and signal transduction in potato does not always resemble what has been described for wild type *A. thaliana*. For example, potato plants have been shown to be hypersensitive to BTH, a chemical analogue of SA (Navarre and Mayo 2004).

Even though an importance of SA signalling in resistance to necrotrophic pathogens has not been indicated for *A. thaliana*, studies using other plant systems, such as oilseed rape (*Brassica napus*) and tomato (*S. lycopersicum)*, have shown the involvement of SA signalling in response to necrotrophic pathogens (Wang et al. 2012; Jia et al. 2013; Nováková et al. 2014). Nováková et al. (2014) investigated defence responses of *B. napus* to the necrotrophic fungus *Sclerotinia sclerotiorum* and found that the amount of SA and expression of SA marker genes were higher in infected plant leaves, which suggest SA involvement in this interaction. Similar results were obtained in tomato, where *NahG* transgenic lines showed increased susceptibility to *Alternaria alternata* (Jia et al. 2013). In addition, the authors found that the JA signalling pathway is also involved in susceptibility since the JA insensitive *jai1* and *spr2* mutants were more resistant, while prosystemin overexpressing plants, that have increased systemin, which triggers JA biosynthesis, were more susceptible to *A. alternata* infection (Jia et al. 2013). In potato, both SA and JA pathways were shown to be required for foliar and tuber defence against the necrotrophic bacterial pathogen *Dickeya solani,* one of the causal agents of blackleg disease (Burra et al. 2015). Additionally, it is becoming increasingly evident that not only the JA and SA signalling pathways are important in the host defence against necrotrophs, but that the plant hormones abscisic acid (ABA) and auxin (IAA) can also modulate host defence against necrotrophs (Mengiste 2012). Hence, a deeper understanding of the genes and hormones involved in pathogen defence in crops is a base to improve cultivar resistance that can be a part of more efficient plant protection strategies.

In this study, we attempt to understand the roles of SA and JA signalling pathways in plant defence response to the necrotrophic pathogen *Alternaria solani* by performing disease bioassays on both potato leaves and tubers, and transcriptomic analysis of infected potato leaves of jasmonate insensitive and salicylic acid-deficient lines in comparison with the control cultivar (cv. Désirée).

## Materials and methods

### Fungal preparation conditions

*Alternaria solani* (isolate AS112), obtained from naturally infected potato plants in Sweden (Odilbekov et al. 2014), was grown on 20% potato dextrose agar medium in 9 cm Petri dishes and incubated in the dark at 25 °C for 7 d. After this, plates were incubated an additional 7 d under UV-c light (model OSRAM HNS15G13 with dominant wavelength 254 nm) for 6 h per day to increase sporulation. The conidia were harvested by flooding the plates with autoclaved tap water containing 0.01% (v/v) Tween 20. The final concentration was adjusted with sterile tap water to 20,000 conidia/ml for the experiments performed in the greenhouse, 25,000 conidia/ml for the growth cabinet experiments, and 10,000 conidia/ml for the tuber bioassay. To ensure that the conidial suspension would stick to the inoculation site on the leaf surfaces, bacto agar was added to a final concentration of 0.033% (w/v).

### Plant materials and growth conditions

*Solanum tuberosum* cv. Désirée (wild type) which is moderately susceptible to *A. solani* (Odilbekov et al. 2014), two transgenic lines (*NahGA, NahGD2*) expressing the *NahG* gene that renders them SA deficient, and two transgenic *coi1* RNAi silenced lines *(coi1H1, coi1X5)* resulting in JA insensitivity (Halim et al. 2004, 2009), were grown in tissue culture in a phyto chamber with 16 h of light (140 μE) at 22 °C for 3 weeks. The in vitro plantlets were transferred to 3.5 L plastic pots filled with commercial pot soil (Yrkesplantjord, Weibulls, Sweden) in a greenhouse chamber with adjusted temperature to 22 °C with 15 h of natural light supplemented with artificial light for the leaf bioassay, fungal biomass measurements and the microarray samples. For the 3,3′-diaminobenzidine (DAB) stained samples, in vitro plantlets of cv. Désirée (wild type), *NahGD2,* and *coi1H1* were transferred to 2 L plastic pots filled with commercial soil (Exclusiv Blom and Plantjord, Emmaljunga Torvmull AB, Sweden) in a biotron chamber for 3.5 weeks (20 °C, 16 h of 160 μmol/m^2^/s light, and 65% humidity). In order to allow acclimatisation and adjustment to the change in environmental conditions compared to the closed in vitro pots, the plantlets were covered with a plastic cup for the first 7 days.

### Fungal inoculations

After 5 weeks in the greenhouse, plants were infected and scored according to Odilbekov et al. (2014). Briefly, an inoculation droplet of 15 μL conidial suspension (20,000 conidia/ml) was placed on the surface of 10 randomly selected leaflets of similar size in the middle part of the plant canopy. For the mock-treatment, plants were drop inoculated with ddH_2_O (double distilled water) containing 0.01% Tween 20 (v/v) and bactoagar 0.033% (w/v). Inoculated plants were kept under a humidity tent in the dark for the first 24 h at a relative humidity (RH) of > 95%. After 24 h, the tent was removed, and the RH was reduced to approximately 75%. The experiment was arranged in three randomised complete blocks and samples were collected at 0, 24, 72 and 120 h post-inoculation (hpi). At each time point, four leaflets were collected from individual plants, pooled and immediately frozen in liquid nitrogen for further experiments. Disease symptom development was determined by measuring the diameter of the lesions 10 dpi. The infection efficiency of the inoculum was also determined at 10 dpi. Statistical analysis comparing the lesion sizes was performed using one-way ANOVA, significance testing was performed using Fisher’s Pairwise comparison’s test (p < 0.05) in Minitab^®^ (Version 18) Statistical Software package (Minitab Inc., 2010). For the DAB stained samples, biotron-grown plants cv. Désirée (wild type), *NahGD2* and *coi1H1* were placed in custom made acrylic glass boxes (422 × 422 × 306 mm) with a tray insert (30 mm high) to allow 1 L water to be placed in the bottom without the plants directly touching the water. When closed the RH inside the box reaches > 95% due to the added water. An inoculation droplet of 10 μL conidial suspension (25,000 conidia/ml) was placed on the surface of as many leaflets in the middle part of plants as possible. The infection boxes were closed and placed in Panasonic versatile environmental test chambers (model MLR-352H-PE) equipped with 15 Panasonic FL40SS ENW/37 lights. The incubators were programmed as follows: 06:00–22:00, 25 °C, 3 lights on, 0 RH; 22:00–06:00, 22 °C, 0 lights on, 0 RH; with the exception of the first 24 h, where the plants were kept in the dark to aid infection, all other conditions were the same. The experiment was arranged in three test chambers with each 3 boxes, 4 plants per box and the plants were placed in the boxes in a completely randomised order. Leaf disc samples 15 mm in diameter were sampled 72 h post-inoculation for DAB staining.

### Complementation experiment

Soil plants grown in the greenhouse for 5 weeks in 3.5 L pots were either watered with 50 ml 1 mM salicylic acid solution or the mock control of tap water 24 h before inoculation with conidial suspension as described in the section above. The 1 mM salicylic acid sodium salt was prepared by dissolving 192 mg of salicylic acid sodium salt (Janssen Chemica) in 1 ml of 100% Ethanol and subsequently adding 1200 ml tap water. Ten days post inoculation the disease symptom development was determined by measuring the diameter of the lesions. Statistical analysis comparing the lesion sizes was performed using one-way ANOVA, significance testing was performed using Fisher’s Pairwise comparison’s test (p < 0.05) in Minitab^®^ (Version 18) Statistical Software package (Minitab Inc., 2010).

### *Alternaria solani* biomass measurements

Four leaflets each from 3 individual plants, were sampled 5 dpi, weighed and powdered using a benchtop tissue homogeniser (FastPrep^®^-24, MPbio, USA). 100 mg of the powder was used to extract genomic DNA using the 1% CTAB method (Doyle 1987). DNA concentration was adjusted to 50 ng/µl and used as template for qPCR. *A. solani* species-specific primer pair OAsF7 (5′ CGACGAGTAAGTTGCCCTCA), and OAsR6 (5′ TGTAGGCGTCAGAGACACCATT) was used, which was designed on the basis of a comparison of the gene Alternaria major allergen *Alt a1* between different *Alternaria* species, as described by (Gannibal et al. 2014). A standard curve of the cycle threshold (Ct) versus fungal dry weight was constructed by performing qPCR on a dilution series of fungal DNA of known fungal dry weight. A conversion factor to correct the effect of differences in leaflet weight was applied. Statistical analysis comparing pathogen biomass amounts was performed using one-way ANOVA, significance testing was performed using Fisher’s Pairwise comparison’s test (p < 0.05) in Minitab^®^ (Version 18) Statistical Software package (Minitab Inc., 2010).

### Tuber bioassay

Wild type (cv. Désirée), *NahGA*, *NahGD2, coi1* × *5* and *coi1H1* tubers stored for 8 months at 4 °C, 80% RH were used for the assay. 8 tubers per plant line were washed with tap water, carefully dried, cut in half, and placed cut side up in a light impermeable plastic box with tap water-soaked cellulose tissue topped with plastic mesh in the bottom to ensure an RH of > 95%. The tuber halves were drop inoculated with 20 μl 10,000 conidia/ml *A. solani* inoculum. The boxes were closed and incubated at 21 °C. At 15 dpi, the horizontal and vertical diameters of the lesions were measured, and the potato halves were halved again to measure the lesion depth. The lesion diameters were averaged and the lesion radius determined. The lesion volume was calculated by approaching the lesion as a sphere cap using the following formula Volume = 1/6πd (3r^2^ + d^2^), where ‘r’ is the lesion radius and ‘d’ the lesion depth (Fig. 2A). Statistical analysis comparing the lesion volumes was performed using one-way ANOVA, significance testing was performed using Fisher’s Pairwise comparison’s test (p < 0.05) in Minitab^®^ (Version 18) Statistical Software package (Minitab Inc., 2010).

### DAB staining

Leaf discs were stained with 3,3′-diaminobenzidine (DAB) to visualise H_2_O_2_ production, according to an adaption of the *Arabidopsis thaliana* protocol by Daubi and O’Brien (2012) for potato leaf discs. Ten leaf discs (15 mm diameter) per plant line were placed in 12-well plates, 2 ml DAB staining solution (1 mg/ml acidified DAB tablets (Sigma D5805), 0.05% Tween-20, 10 mM Na_2_HPO_4_) was added to each well followed by incubation at RT for 4 h on 40 rpm shaker. Leaf discs were subsequently destained in 2 ml destaining solution (3:1:1 Ethanol (100%): Acetic acid: Glycerol) in an 85 °C incubator for 20 min. The warm destaining solution was removed and 100% EtOH added, and the leaves were placed on the RT shaker overnight and analysed the next day or kept at 4 °C for up to 4 d. Pictures of the stained leaf discs were taken with a Leica stereomicroscope M165FC with DFC 450C camera attached using the LAS v1.12 software. The level of DAB staining was analysed using the image analysis software MIPAR™ v2.1.9 (Sosa et al. 2014). All images were cropped to the same size avoiding edges of the leaf discs and subsequently colour segmented for the DAB staining. The segmented image was binarised, and the DAB area fraction was calculated. The analysis was automated in a MIPAR recipe with an interruptible crop step to ensure no leaf disc edges were included in the new image, as well as an interruptible segmentation step to manually adjust incorrectly segmented objects if needed. Statistical analysis comparing the DAB area fraction was performed using one-way ANOVA, significance testing was performed using Fisher’s Pairwise comparison’s test (p < 0.05) in Minitab^®^ (Version 18) Statistical Software package (Minitab Inc., 2010).

### RNA extractions and microarray preparations

RNA was extracted from cv. Désirée (wild type), *NahGD2,* and *coi1H1* plants, either mock or *A.solani* inoculated, sampled at either 24, 72 or 120 hpi. For each sample, 4 leaflets from the same plant were pooled, frozen in liquid nitrogen and ground into a fine powder using a pre-cooled mortar and pestle. RNA was isolated from 100 mg of frozen tissue using the Qiagen RNeasy mini kit (Qiagen, Hilden, Germany) according to the manufacturer’s protocol. RNA concentration and purity were determined by ND-1000 NanoDrop (Wilmington, USA). Subsequently, the integrity of the samples was corroborated using the Experion™ Automated Electrophoresis System (Bio-Rad Laboratories, Hercules, USA). All RNA samples were adjusted to 200 ng/μl and microarray analysis was performed using a custom Agilent microarray designed to the predicted transcripts from of the *Solanum tuberosum* Group Phureja DM genome (assembly v.3.4) as described previously (Hancock et al. 2014). A single-channel microarray design was used, and the experimental design and data are available (ArrayExpress: https://www.ebi.ac.uk/arrayexpress/; accession E-MTAB-8477). RNA labelling and microarray processing were performed as recommended by the manufacturer (One-Color Microarray-Based Gene Expression Analysis protocol v.6.5; Agilent) using the Low Input Quick Amp Labelling kit (Agilent). Microarrays were scanned using an Agilent G2505B scanner with images processed using Feature Extraction (v.10.7.3.1) software, aligned with the corresponding array grid (033033_D_F_20110315) and extracted with the FE protocol (GE1_107_Sep09).

### Microarray data analysis

The raw microarray data were imported and analysed in R studio (version 3.3.2 ©2016) using the Bioconductor LIMMA (Linear Models for Microarray and RNA-Seq Data) package (Ritchie et al. 2015). The data was read into R, guided by a target file that listed the correct filenames for the different arrays, conditions, treatment and sampling time. The raw intensity values of each array were corrected for background fluorescence. The values were subsequently normalised between arrays using the quantile method. Replicate probe values were averaged, and a new value list was produced with average intensity values for the probes, and subsequently a linear model was fitted to each probe to determine the fold changes and standard errors. To analyse the changes in gene expression for a specific time point between mock and infected plants, contrast matrices were constructed for the *Alternaria solani* infected arrays compared to mock arrays of the same genotype at the same time point, the intensity values were fitted with a linear model and further processed to produce empirical Bayes test statistics for each probe, including moderated t-statistics, p-values and fold-changes as log2 fold change in expression. Probes with an adjusted p-value < 0.05 were extracted and subsequently subsetted into upregulated, if log2 Fold Change (FC) > 0, or downregulated, if log2 FC < 0. ProbeIDs were annotated with the *S. tuberosum* group Phureja DM1-3 516 R4 (DM) gene model numbers (DMG) by merging the new tables with the PLAZA 4.0 gene description annotation file (The Potato Genome Sequencing Consortium 2011). The webtool BioVenn (Hulsen et al. 2008) was used to analyse the overlapping and unique differentially expressed genes (DEGs) of the different genotypes at the three different time points. Venn diagrams were created using meta-chart.com/venn. Gene Ontology terms were assigned to the DEGs by using the locus ID v3.4 (Phytozome v11.0) annotation downloaded from AgriGo v2.0 (Tian et al. 2017). In the case of more than one probe annotated with the same DMG number appearing in the list of DEGs, the probe with the highest log FC was used for GO (Gene Ontology) analysis, in order to avoid the same DMG number appearing in the list multiple times. Singular Enrichment Analysis (SEA) was performed at the AgriGO v2 website (https://systemsbiology.cau.edu.cn/agriGOv2/), using the locus ID v3.4 (Phytozome v11.0) as background reference, to find significantly enriched (FDR < 0.05) GO terms using standard settings. The bubble plot displaying the significantly enriched GO terms for the DEGs in the infected *NahG* plant compared to the mock-inoculated plants at 120 hpi was created using the R-package GOplot (Walter et al. 2015).

## Results and discussion

### Larger lesions develop on SA deficient plants

**Fig. 1:**
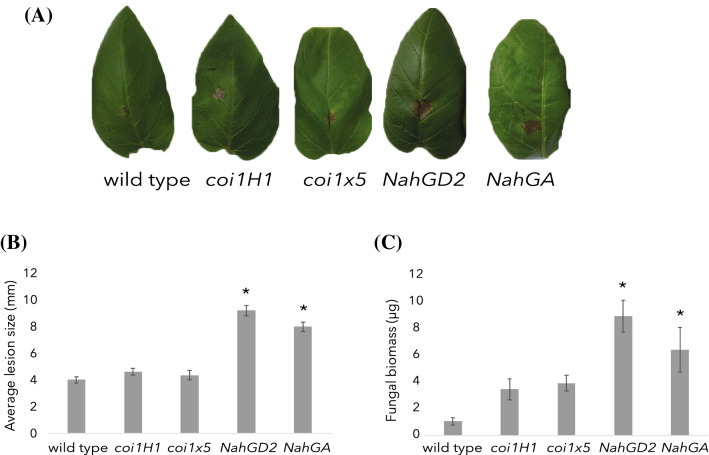
Salicylic acid-deficient potato plants (*NahGD2* and *NahGA*) show larger leaf lesions and higher fungal biomass after *Alternaria solani* infection than wild type plants. Representative images (**A**) and average lesion size (mm) (**B**) at 10 days post-inoculation (dpi) for one wild type (N = 57 leaflets) and four mutant lines, two jasmonic acid insensitive (*coi1H1* (N = 54 leaflets) and *coi1* × *5* (N = 35 leaflets)) and two salicylic acid-deficient (*NahGD2* (N = 63 leaflets) and *NahGA* (N = 59 leaflets)) lines. Both SA deficient lines showed significantly larger lesions compared to wild type. The fungal biomass (**C**) in μg/100 mg fresh weight harvested 5dpi determined by qPCR N = 3. Both SA deficient lines show significantly higher fungal biomass compared to wild type. Error bars represent standard error of the mean. Asterisks represent significant differences in comparison to wild type as tested by one-way ANOVA followed by Fisher’s Pairwise comparison’s test (p < 0.05)

In order to investigate the role of salicylic acid (SA) and jasmonic acid (JA) in disease symptom development due to *Alternaria solani* infection on potato leaves, a foliage bioassay was performed. Leaflets of whole plants of wild type (cv. Désirée), two JA insensitive (*coi1H1 and coi1* × *5*) and two SA deficient (*NahGD2 and NahGA*) lines were drop inoculated with *A. solani* conidia inoculum and the lesion size measured ten days post-inoculation (dpi). Surprisingly, the SA deficient lines showed significantly larger disease lesions compared to the wild type plants and the JA insensitive lines (Fig. 1A, B). No significant difference between wild type and JA insensitive plants was detected. To test whether the larger lesions were due to a reaction of the plant or growth of the fungus, fungal biomass was determined using qPCR of the Alternaria major allergen *Alt a1* gene. The fungal biomass was significantly higher in the SA deficient lines compared to the wild type (Fig. 1C), indicating that the visible lesion follows the growth of the pathogen. In order to determine whether the increased lesion size in the SA deficient plants can be reverted by SA, we performed SA soil application experiments followed by inoculation with *A. solani*. Both the SA deficient lines (*NahGD2* and *NahGA*) showed significant reduction in lesion size when the plants were watered with SA 24 h before inoculation compared to the mock drenched control plants (online resource 1). The SA treated plants show similar *Alternaria* lesion sizes as wild type, indicating that the absence of SA results in observed enhanced susceptibility of the *NahG* plant lines to *A. solani*.

### Tubers from SA deficient plants had larger early blight lesions

**Fig. 2:**
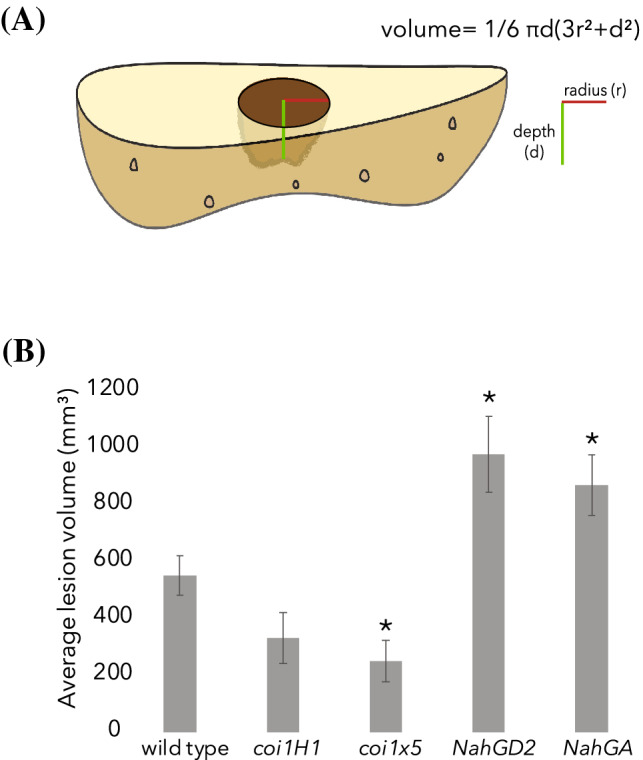
Salicylic acid-deficient potato tubers (*NahGD2* and *NahGA*) show larger lesions 15 days after *Alternaria solani* infection than wild type tubers. Schematic figure of *A. solani* tuber lesion and the formula used to calculate the lesion volume (**A**) and average lesion size volume (mm^3^) (**B**) at 15 days post-inoculation (dpi) for one wild type and four mutant lines, two jasmonic acid insensitive (*coi1H1* and *coi1* × *5*) and two salicylic acid-deficient (*NahGD2* and *NahGA*) lines (N = 16). Both SA deficient lines showed significantly larger lesions compared to wildtype. Error bars represent standard error of the mean. Asterisks represent significant differences in lesion volume in comparison to wildtype as tested one-way ANOVA followed by Fisher’s Pairwise comparison’s test (p < 0.05)

Since *A. solani* does not only affect the foliage but can also infect the potato tubers, a tuber bioassay was performed to study the disease development in the wild type and hormone compromised plants. The lesion volume was calculated based on the radius and depth measurements by approaching the shape of the lesion as a spherical cap (Fig. 2A). Comparable to the leaf bioassay, the SA deficient tubers had significantly larger lesion volumes than wild type tubers (Fig. 2B).

The larger leaf and tuber lesions and higher fungal biomass in *NahG* plants compared to infected wild type plants, indicate that intact SA signalling is necessary to mount an efficient defence response to *A. solani* in both foliage and tubers. Our finding of larger leaf lesions in the *NahG* plants is similar to results obtained by Jia et al. (2013) who observed that tomato *NahG* plants had larger lesion size and higher pathogen biomass in comparison to wild type upon infection with *Alternaria alternata.* Relatively little is known about the role of phytohormones in defences against pathogens attacking tubers. Thangavel et al. (2016) showed that more common scab-resistant tubers had higher levels of suberin biosynthesis and that suberin within the periderm is crucial for repairing damage and preventing penetration by fungi and bacteria. One of the enzymes required for the biosynthesis of suberin is phenylalanine ammonia-lyase (PAL), an enzyme that is also involved in the synthesis of SA through the chorismate pathway. High PAL activity may to be important for pathogen-triggered SA biosynthesis in several plant systems (Chen et al. 2009).

Moreover, a previous study also showed that SA induces resistance to *A. solani* in tomato leaves and that this is mediated by systemic acquired resistance signals (Spletzer and Enyedi 1999). Additionally, Sarkar et al. (2017) found that SA biosynthesis pathways were enriched in tomato leaves 3 days after infection with *A. solani*. In contrast, another study found that detached tomato leaflets sprayed with SA were more susceptible to *A. solani* compared to control leaflets (Rahman et al. 2012). These opposing results could be explained by use of detached leaflets by Rahman et al. (2012) compared to use of whole plants in the other studies, including this one. We have previously shown that the *A. solani* response obtained from detached leaflet assays and field assays do not correlate in potato, but that whole-plant assays as carried out in the present study are in line with field data (Odilbekov et al. 2014). The results of the current study do not exclude the hypothesis that the observed SA mediated resistance to *A. solani* could be dependent on systemic acquired resistance signals, as proposed by Spletzer and Enyedi (1999). Consequently, if this is so, this further strengthens the case that detached leaf assays are not an appropriate method to assess the role of defence signalling in potato defences against *A. solani*. In summary, contradictory to the majority of the studies in *Arabidopsis thaliana* in which a more prominent role of JA has been proposed in resistance to necrotrophic pathogens (Glazebrook 2005), we observed the importance of intact SA signalling in potato defence responses to the necrotrophic fungus *A. solani*.

### Less H_2_O_2_ is produced in SA deficient and JA insensitive plants than in the wild type

**Fig. 3:**
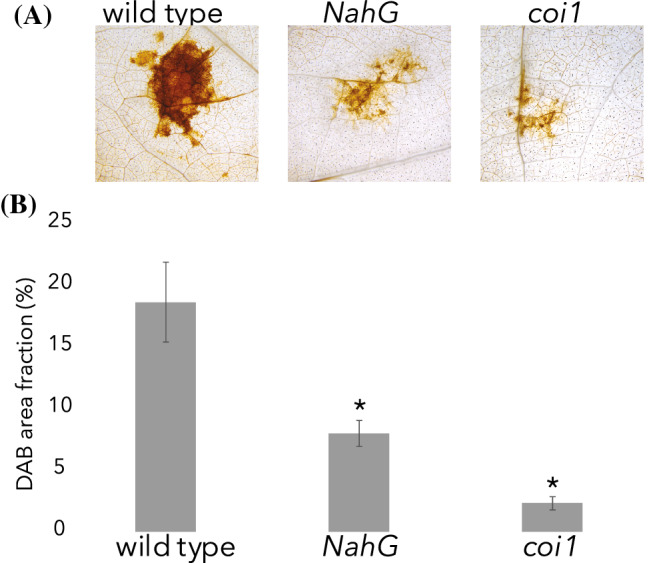
Salicylic acid deficient and jasmonic acid insensitive lines produce less H_2_O_2_ in response to *A. solani* infection than wild type. DAB (3,3–diaminobenzidine) stained area fraction of *A. solani* infected area. Representative images (**A**) and average area fraction in % of brownish DAB staining (**B**) at 72 hpi, wild type, JA insensitive (*coi1H1*), and SA deficient (*NahGD2*) lines. Error bars represent standard error of the mean (N = 20). Asterisks represent significant differences in comparison to wild type as tested by one-way ANOVA followed by Fisher’s Pairwise comparison’s test (p < 0.05)

The effect of *A. solani* infection on the oxidative burst was determined by visualising the presence of hydrogen peroxide (H_2_O_2_) using 3,3-diaminobenzidine (DAB) staining. Leaf discs containing an *A. solani* infected area were harvested 72 dpi and stained in DAB and subsequently de-stained. Both hormone compromised lines showed significantly smaller areas of staining, corresponding to H_2_O_2_ producing areas, compared to the wild type (Fig. 3).

### Transcriptional changes are induced due to *A. solani* infection

**Fig. 4:**
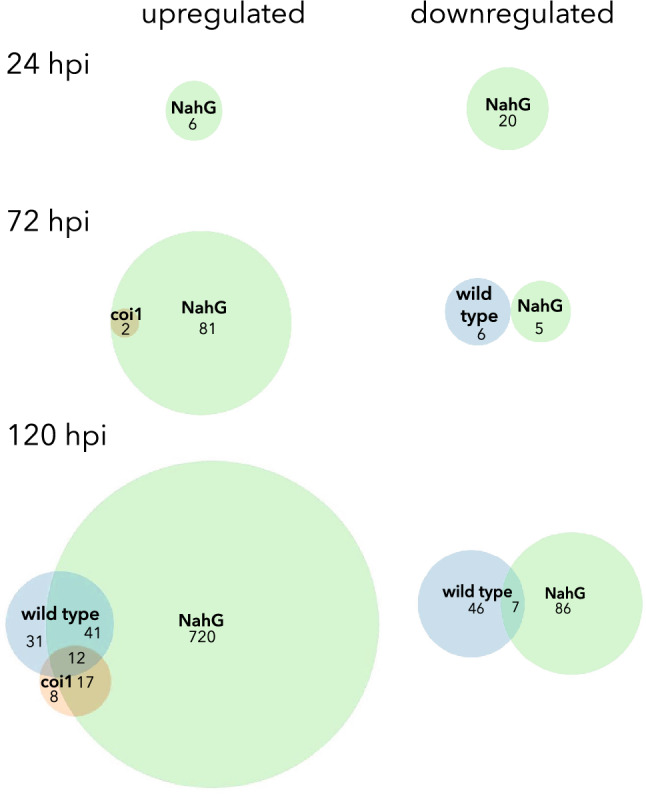
Venn diagrams depicting the overlap of the total the number of significantly upregulated and downregulated genes (adj. p value < 0.05) at 24, 72 and 120 hpi in the wild type, jasmonic acid insensitive (*coi1H1*) and salicylic acid-deficient (*NahGD2*) lines

The response of potato gene expression to *A. solani* infection*,* and the role herein of salicylic acid (SA) and jasmonic acid (JA), was further explored by a transcriptome analysis by microarray of wild type, *NahGD2* and *coi1H1* plants at three time points after inoculation. The whole data set with 36 arrays has been deposited in the ArrayExpress database at EMBL-EBI (www.ebi.ac.uk/arrayexpress) under accession number E-MTAB-8477. We decided to use complete leaflets to study the effect of infection, since hormones are likely to be important close to the inoculation site and also systemically. Samples for the microarray experiment were successfully infected since the infection efficiency of the inoculum was determined at 10 dpi to be over 85% for all genotypes. The number of differentially expressed genes (DEGs) between *A. solani* and mock-inoculated samples at the same time point for all three plant types is presented in Table 1. The top 10 DEGs for each plant line at the different time points are presented in Table 2. The complete list of DEGs is presented in online resource 2, for some of the DEGs, multiple probes are linked to the same gene, for these genes all probes are presented in online resource 2. The overlap between the DEGs between the wild type, SA deficient and JA insensitive plant lines at the 3 different timepoints is visualised in Venn diagrams (Fig. 4).

**Table 1:** Number of differentially expressed genes (DEGs) upon infection with *A. solani* in the wild type (cv. Désirée), JA insensitive (*Coi1H1)* and SA deficient (*NahGD2)* plants at 24, 72, and 120 h post-inoculation (hpi)

	Upregulated			Downregulated		
	Wild type	JA insensitive	SA deficient	Wild type	JA insensitive	SA deficient
24 hpi	0	0	6	0	0	20
72 hpi	0	2	83	6	0	5
120 hpi	84	37	790	53	0	93

**Table 2:** Top 10 differentially expressed genes (DEGs) upon infection with *A. solani* compared to the control for wild type (cv. Désirée), JA insensitive (*coi1H1*), and SA deficient (*NahGD2*) plant lines at 24, 72 and 120 hpi. Gene name (DMG), gene description and the Log2 Fold change of infected compared to control are displayed

24 hpi	SA deficient	
Gene name (DMG)	Gene description	Log2 fold change
PGSC0003DMG400010839	Aromatic amino acid decarboxylase 1B	3.11
PGSC0003DMG400026382	Gamma-glutamyl-gamma-aminobutyrate hydrolase	1.96
PGSC0003DMG400015536	MYB1-2	1.80
PGSC0003DMG400000952	Phenazine biosynthesis protein	− 3.07
PGSC0003DMG400000951	Phenazine biosynthesis protein	− 3.07
PGSC0003DMG400042670	Gene of unknown function	− 2.66
PGSC0003DMG400011710	Conserved gene of unknown function	− 2.63
PGSC0003DMG400034948	Gene of unknown function	− 2.04
PGSC0003DMG400036005	Gene of unknown function	− 1.86
PGSC0003DMG400013187	Hexokinase 6	− 1.85

72 hpi	Wild type	
Gene name (DMG)	Gene description	Log2 fold change
PGSC0003DMG400003367	Conserved gene of unknown function	− 2.24
PGSC0003DMG400017064	Gene of unknown function	− 2.34
PGSC0003DMG400020672	Conserved gene of unknown function	− 2.89
PGSC0003DMG400020673	Conserved gene of unknown function	− 2.50
PGSC0003DMG400022246	Polyubiquitin	− 1.40
PGSC0003DMG400040313	Gene of unknown function	− 1.41

	JA insensitive	
PGSC0003DMG400029201	Sesquiterpene synthase 2	3.37
PGSC0003DMG400013763	Ankyrin repeat-containing protein	2.09

	SA deficient	
PGSC0003DMG400010859	Lipoxygenase	4.17
PGSC0003DMG400018916	Polyphenol oxidase	4.13
PGSC0003DMG400031849	2-Hydroxyisoflavanone dehydratase	3.60
PGSC0003DMG400040260	Glucan endo-1,3-beta-glucosidase	3.33
PGSC0003DMG402029631	Pleiotropic drug resistance protein 1	3.26
PGSC0003DMG400003058	Osmotin OSML15	3.23
PGSC0003DMG400029562	Cytochrome P450	3.13
PGSC0003DMG400003993	Citrate binding protein	3.08
PGSC0003DMG400023435	Major allergen Pru ar	3.07
PGSC0003DMG400020017	Lichenase	2.95

120 hpi	Wild type	
Gene name (DMG)	Gene description	Log2 fold change
PGSC0003DMG400026222	Major pollen allergen Ory s 1	5.26
PGSC0003DMG400023922	Cytoplasmic small heat shock protein class I	4.47
PGSC0003DMG400029201	Sesquiterpene synthase 2	4.42
PGSC0003DMG400010283	Class I chitinase	3.98
PGSC0003DMG400001550	TSI-1 protein	3.74
PGSC0003DMG400002027	Cytoplasmic small heat shock protein class I	3.61
PGSC0003DMG400002028	Cytoplasmic small heat shock protein class I	3.60
PGSC0003DMG400021109	Conserved gene of unknown function	3.58
PGSC0003DMG400014702	Conserved gene of unknown function	3.39
PGSC0003DMG400029830	Glucan endo-1,3-beta-d-glucosidase	3.30

	JA insensitive	
PGSC0003DMG400029201	Sesquiterpene synthase 2	3.81
PGSC0003DMG401024842	Conserved gene of unknown function	3.78
PGSC0003DMG400023922	Cytoplasmic small heat shock protein class I	3.08
PGSC0003DMG401008167	AT-HSFB3	2.93
PGSC0003DMG400002028	Cytoplasmic small heat shock protein class I	2.88
PGSC0003DMG400002027	Cytoplasmic small heat shock protein class I	2.86
PGSC0003DMG400022929	Aspartate aminotransferase	2.73
PGSC0003DMG400001550	TSI-1 protein	2.70
PGSC0003DMG400013469	Conserved gene of unknown function	2.61
PGSC0003DMG400001948	Copalyl diphosphate synthase	2.58

	SA deficient	
PGSC0003DMG400019435	Wound-induced protein WIN1	8.22
PGSC0003DMG400003044	Osmotin	7.12
PGSC0003DMG400010859	Lipoxygenase	7.04
PGSC0003DMG400003993	Citrate binding protein	6.84
PGSC0003DMG400037874	PR1 protein	6.74
PGSC0003DMG400043736	PR1 protein	6.67
PGSC0003DMG400010859	Lipoxygenase	6.64
PGSC0003DMG400040260	Glucan endo-1,3-beta-glucosidase	6.58
PGSC0003DMG400005115	PR1 protein	6.55
PGSC0003DMG400005116	PR1 protein	6.49

### SA deficient plants show more differentially expressed transcripts than the wild type

The number of differentially expressed genes increased with time after infection. At 24 hpi the only differentially expressed were 26 genes in SA deficient plants (Table 1 and Online resource 2). Overall, the SA deficient plants showed the highest number of DEGs, especially among the upregulated DEGs, while very few DEGs were found for the JA insensitive plants (Table 1). All genes that were upregulated in the JA insensitive plants at 72 hpi were also up in the SA deficient plants. However, the genes downregulated at 72 hpi in the wild type and SA deficient do not show any overlap. At 120 hpi we find the most DEGs, with SA deficient plants having the highest total number of DEGs and the highest number of unique DEGs. A set of 12 genes is upregulated in all genotypes (Fig. 4 and Online Resource 3). Among these 12 genes, we find three genes encoding cytoplasmic small heat shock proteins belonging to class I (PGSC0003DMG400002027, PGSC0003DMG400002028, PGSC0003DMG400023922). Heat shock proteins (HSPs), are known to be induced by both abiotic and biotic stresses and play an important role in plant immunity by acting as molecular chaperones of both membrane and intracellular receptor proteins (Park and Seo 2015). Another shared gene is annotated as encoding a TSI-1 protein, the protein sequence shows high similarity (98% identity) to pathogenesis induced protein STH-2 in potato. To analyse if the large upregulation in the SA deficient plants at 72 hpi (83 genes) only reflects larger or faster development of lesions, we compared these genes with the similar number at 120 hpi for the wild type (84 genes). However, the majority of the genes upregulated in the SA deficient plants at 72 hpi and the wild type at 120 hpi did not overlap (~ 80%). Only 17 genes (~ 20%) were found to be upregulated in both.

### GO enrichment analysis

**Fig. 5:**
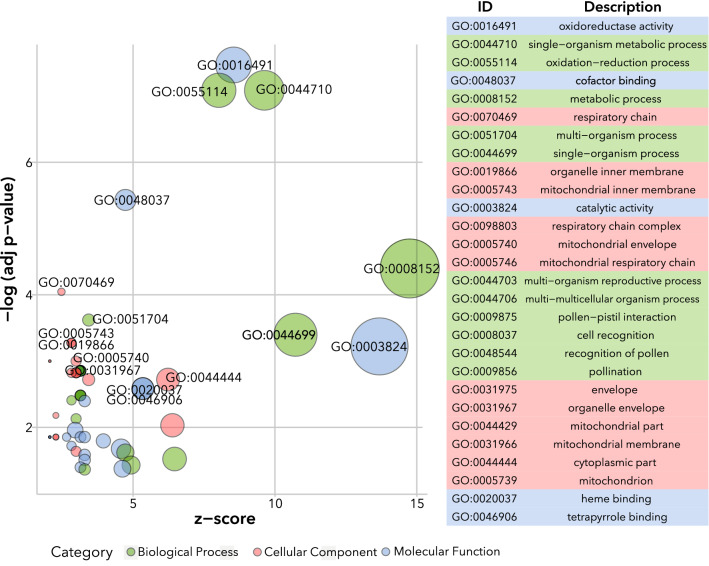
Bubble plot showing significantly enriched Gene Ontology (GO) terms for the differentially expressed genes (DEGs) in *A. solani* infected SA defiencient plant line (*NahGD2*) versus control at 120 hpi. Size of the bubbles is proportional to the number of DEGs (adj. p value < 0.05) assigned to the GO term. The y-axis represents the negative logarithm of the adjusted p value [false discovery rate (FDR)] for the GO terms, and the x-axis displays the z-score as calculated using the GOplot R-Package (Walter et al. 2015). The threshold for displaying the bubble labels was set to a − log(FDR) of 2.5. Bubbles for GO terms belonging to Biological Process are green, Molecular Function are blue, and Cellular Component are red. Gene name (DMG), gene description and the Log2 Fold change in the infected compared to the control are displayed.

In order to gain a better understanding of the relationships and function of the differentially expressed genes, a GO (Gene Ontology) enrichment analysis was performed (online resource 4). For the wild type, significantly enriched GO terms could only be found for the DEGs at 120 hpi (Table 3). At this timepoint wild-type plants show significant upregulation of genes classed into GO terms related to cation, ion, substrate-specific, and substrate-specific transmembrane transporter activity, cofactor binding and hydrolase activity (Table 3). In the SA deficient plants, the DEGs at 72 hpi showed enrichment of the GO terms cofactor binding and oxidoreductase activity (Table 4). At 120 hpi in the SA deficient plants, both these GO terms are still significantly enriched and are among the most highly enriched GO terms (Fig. 5, Online resource 4). Other highly enriched GO terms are single-organism metabolic process (GO:0044710), metabolic process (GO:0008152), single- and multi-organisms process (GO:0044699, GO:1704005). When comparing the significantly enriched GO terms of the wildtype and NahG SA deficient plants at 120 hpi, only GO:0048037 (cofactor binding) is significantly enriched in both. No significantly enriched GO terms were found for the DEGs in the JA insensitive plants or in the core group of 12 genes that are upregulated in all three genotypes at 120 hpi.

**Table 3:** Significantly enriched Gene Ontology (GO) terms with their associated differentially expressed genes (DEGs) in *A. solani* infected wild type plants (cv. Désirée) versus control at 120 hpi

GO term	Gene name (DMG)	Gene description	Log2 Fold change
and GO:0022891 Substrate-specific transmembrane transporter activity GO:0022892, Substrate-specific transporter activity GO:0015075, Ion transmembrane transporter activity GO:0008324, Cation transmembrane transporter activity GO:0022890, Inorganic cation transmembrane transporter activity	PGSC0003DMG400020829	Copper transporter 1	2.97
	PGSC0003DMG400010373	Iron-regulated transporter 1	1.67
	PGSC0003DMG400002151	Zinc transporter	1.34
	PGSC0003DMG400017913	Cytochrome-c oxidase	0.74
	PGSC0003DMG402030815	Cytochrome C oxidase polypeptide vib	0.70
	PGSC0003DMG400026508	ATP synthase epsilon subunit 1	0.54
GO:0015075, GO:0022892 and GO:0022891	PGSC0003DMG400009469	Sulfate transporter 2	0.94
GO:0004553 Hydrolase activity, hydrolyzing O-glycosyl compounds	PGSC0003DMG400029830	Glucan endo-1,3-beta-D-glucosidase	3.30
	PGSC0003DMG400040260	Glucan endo-1,3-beta-glucosidase, basic isoform 1	2.86
GO:0016798 Hydrolase activity, acting on glycosyl bonds	PGSC0003DMG400020017	Lichenase	2.82
	PGSC0003DMG400001528	Class II chitinase	1.90
	PGSC0003DMG400010490	Acidic class II 1,3-beta-glucanase	1.86
	PGSC0003DMG402001531	Chitinase 134	1.63
	PGSC0003DMG401010492	Acidic class II 1,3-beta-glucanase	1.45
GO:0048037 Cofactor binding	PGSC0003DMG400021109	Conserved gene of unknown function	3.58
	PGSC0003DMG400022929	Aspartate aminotransferase	2.85
	PGSC0003DMG400030082	Primary amine oxidase	2.47
	PGSC0003DMG400033906	Isoflavone reductase homolog	1.98
	PGSC0003DMG400007059	RHM1/ROL1	− 1.26
	PGSC0003DMG400006270	Pyruvate decarboxylase	− 2.16
	PGSC0003DMG400021142	DWARF1/DIMINUTO	− 2.44

**Table 4:** Significantly enriched Gene Ontology (GO) terms with their associated differentially expressed genes (DEGs) in *A. solani* infected SA defiencient plant line (*NahGD2*) versus control at 72 hpi. Gene name (DMG), gene description and the Log2 Fold change in the infected compared to the control are displayed

GO term	Gene name (DMG)	Gene description	Log2 Fold Change
GO:0048037 Cofactor binding	PGSC0003DMG400030082	Primary amine oxidase	2.82
	PGSC0003DMG400000193	1-aminocyclopropane-1-carboxylate synthase 2	2.35
	PGSC0003DMG400022929	Aspartate aminotransferase	2.06
	PGSC0003DMG400000496	Formate dehydrogenase, mitochondrial	2.02
	PGSC0003DMG400026801	Formate dehydrogenase	1.84
	PGSC0003DMG400003461	3-hydroxy-3-methylglutaryl coenzyme A reductase	1.56
	PGSC0003DMG400027919	Aspartate aminotransferase	0.79
GO:0016491 Oxidoreductase activity	PGSC0003DMG400010859	Lipoxygenase	4.17
	PGSC0003DMG400018916	Polyphenol oxidase	4.13
	PGSC0003DMG400029562	Cytochrome P450	3.13
	PGSC0003DMG400030082	Primary amine oxidase	2.82
	PGSC0003DMG400020334	Prephenate dehydrogenase	2.43
	PGSC0003DMG400025158	Divinyl ether synthase	2.42
	PGSC0003DMG400013879	Quinone reductase family protein	2.27
	PGSC0003DMG400020809	Cytochrome P450	2.07
	PGSC0003DMG400000496	Formate dehydrogenase, mitochondrial	2.02
	PGSC0003DMG400026801	Formate dehydrogenase	1.84
	PGSC0003DMG400004800	Gene of unknown function	1.63
	PGSC0003DMG400003461	3-hydroxy-3-methylglutaryl coenzyme A reductase	1.56
	PGSC0003DMG400028175	Cytochrome P450 76A2	1.36

### DEGs are associated with secondary metabolism and cell death at 24 hpi in SA deficient plants

At 24 hpi, we only detected DEGs in the SA-deficient plants, with twenty-six genes differentially regulated. Among the upregulated genes, several play roles in secondary metabolism and one gene is a conserved gene of unknown function (Table 2 and Online resource 2). PGSC0003DMG400029243 is annotated as encoding P-coumaroyl quinate/shikimate 3′-hydroxylase (StC3′H); this enzyme is part of the chlorogenic acid (CGA) biosynthesis pathway (Knollenberg et al. 2018). CGA functions as an antioxidant, and tomato plants with transgenic elevated levels of CGA showed slower disease progression by *Pseudomonas syringae* (Niggeweg et al. 2004). In potato, CGA together with other phenolics plays a positive role in defence against the necrotrophic soft rot pathogen *Erwinia carotovora* (Ghanekar et al. 1984). Interestingly, this gene is the only gene out of the 20 upregulated genes at 24 hpi that is also upregulated at a later time point (120 hpi). Another upregulated secondary metabolism-related gene is annotated as encoding aromatic amino acid decarboxylase 1B (Table 2) and it’s homolog in tomato, is involved in the synthesis of the antimicrobial phenylalanine derived compound 2-phenylethanol (Tieman et al. 2006). Another upregulated gene is annotated as encoding a Gamma-glutamyl-gamma-aminobutyrate hydrolase (Table 2). This is a homolog of the *A. thaliana* GAT1-like protein (AT5G38200), a glutamine amidotransferase protein, and the *Capsicum annuum* Ncn7004 (Bae et al. 2013). Bae et al. (2013) performed virus-induced silencing in *Nicotiana benthamina* and showed that when infected with an avirulent pathogen, the Ncn7004 silenced plants display delayed HR and, when infected with a virulent strain, the development of disease symptoms was delayed compared to the silencing control. It is suggested that the GAT1-like protease plays a role in developmental and pathogen-induced cell death, such as HR. In our study, we only find differential expression of Gamma-glutamyl-gamma-aminobutyrate hydrolase in the SA-deficient plants at 24 hpi (Table 2 and Online resource 2). In this case, the upregulation of the gene could lead to increased pathogen-induced cell death. Increased cell death in the SA-deficient plants at this early time point could potentially be linked to larger lesions detected in the SA deficient plants compared to the wild type since, due to the necrotrophic lifestyle of *A.solani,* the pathogen would benefit from the induced cell death (Govrin and Levine 2000). Indeed, *Alternaria alternata*, and other necrotrophic fungi, are known to produce sphinganine analog mycotoxins (SAMTs) which induce cell death in plants (Shao et al. 2020). A gene that is only differentially expressed at 24 hpi in SA deficient plants, is a gene annotated as a breast carcinoma amplified sequence (PGSC0003DMG400019227), the encoded protein shares homology with *Autophagy related gene* 18f (*ATG18f*) and is found in several species, such as *Solanum pennellii, S. lycopersicum,* and *A. thaliana*. Autophagy related genes (ATG) have been described to be involved in responses to biotic stress (Slavikova et al. 2008; Lai et al. 2011). *A. thaliana* mutants lacking *ATG18a* showed increased spreading of necrosis when infected with *Alternaria brassicicola,* compared to the wild type, suggesting that this autophagy-related gene in *A. thaliana* is important for restricting the development of disease lesions (Lenz et al. 2011). Wang et al. (2016) showed that SA promotes autophagy in *A. thaliana* for senescence and immune responses, however, the endogenous SA levels did not appear to play a role in this. Although it is thought that SA and autophagy influence each other to cause cell death, the mechanism(s) of this interaction remain one of the biggest outstanding questions in the field of plant autophagy research (Leary et al. 2019). Given that we only found this gene differentially expressed at 24 hpi in the SA-deficient plants that develop larger lesions, this could indicate that this gene plays a role in the regulation of cell death. This, however, remains to be tested.

### Ubiquitin is downregulated at 72 hpi in wild type

The infection of *A. solani* in the wild type does not result in differential gene expression, until 72 hpi in the intact leaflet. At this time point, six genes are downregulated, five out of these six genes are annotated as genes of unknown function and do not have any homology to characterised genes or proteins in other species (Table 2). The other gene that is downregulated encodes polyubiquitin (Table 2). A homolog of this gene in *A. thaliana* (AT4G05050) encodes UBQ11 polyubiquitin that shows upregulation during growth and development, with particularly high expression during senescence and in the mature flower state (Klepikova 2016). Downregulation of this gene is observed during infection with the obligate biotrophic oomycete *Hyaloperonospora arabidopsidis* (Wang et al. 2011). Contrastingly, during inoculation with the necrotrophic fungus *Botrytis cinerea, A. thaliana* shows upregulation of the UBQ11 gene; however, the upregulation is observed at earlier time points (Data from Ausubel Lab and Scheel Lab deposited in the Arabidopsis eFP browser; *Winter et al. 2007). A possible outcome for the downregulation of this gene in *A. solani* infected potato could be to put more energy into defence, and limit plant senescence since such tissue is more susceptible to *A. solani* infection (Odilbekov et al. 2014).

### DEGs associated to oxidoreductase activity are upregulated in SA deficient plants at 72 hpi

At 72 hpi, we detected an extensive increase in the number of DEGs in the SA deficient plants (*NahGD2*), 99 genes were upregulated and 5 down regulated. The upregulated ones are significantly enriched in two GO terms GO:0016491 oxidoreductase activity and GO:0048037 cofactor binding (Table 4). The most upregulated gene encodes a Lipoxygenase (LOX), associated to GO: 0016419 (Table 2 and 4). LOX genes are well known to play a role in plant responses to abiotic and biotic stresses. The LOX enzymes are part of the biosynthesis of many oxylipins with crucial roles in plant defences, such as jasmonic acid (Vick and Zimmerman 1983; Blée 2002). Three other genes annotated as cytochrome P450 were also associated to this GO term (Table 4). Enzymes from the cytochrome P450 superfamily play an important role in promoting plant growth and defences. (Xu et al. 2015). Another upregulated gene encodes a quinone reductase family protein (PGSC0003DMG400013879). In *A. thaliana* quinone reductases have been shown to have a role in host-fungus interactions. Overexpression of quinone reductases resulted in hypersensitivity to the necrotrophic fungi *Botrytis cinerea* and *Sclerotinia sclerotium*, whereas knockout lines showed reduced susceptibility and higher ROS levels (Heyno et al. 2013). L’Haridon et al. (2011) previously showed that exogenous H_2_O_2_ application, as well as H_2_O_2_ produced by *A. thaliana* in response to wounding, can enhance resistance to *B. cinerea.* Interestingly, we find the quinone reductase family gene upregulated in the SA deficient plants at 72 hpi and 120 hpi, and in the JA insensitive plants at 120 hpi, but not in the wild type. In our DAB staining experiments, both of the hormone compromised lines showed significantly reduced levels of H_2_O_2_ compared to the wild type (Fig. 3), which does not show upregulation of this gene. However, in our study only the SA deficient lines show significantly larger lesions and increased fungal biomass. The JA insensitive lines show a reduction in H_2_O_2_ but no significant difference in lesion size or fungal biomass compared to the wild type. The other enriched GO term at 72 hpi in the SA deficient plants, GO:0048037 cofactor binding, encompasses two aspartate aminotransferase genes (Table 4). The homolog of one of these genes (PGSC0003DMG400027919) in *A. thaliana* (AT2G22250) encodes a prephenate aminotransferase (PAT), involved in the aromatic amino acid pathway. This is an important pathway involved in the biosynthesis of many aromatic secondary metabolites (Graindorge et al. 2010). Another upregulated gene encoding 3-hydroxy-3-methylglutaryl coenzyme A reductase (HMGR) (PGSC0003DMG400003461), was shown to catalyse the first step of the mevalonate pathway for isoprenoid biosynthesis. This pathway supplies many precursors for products with several functions, including phytoalexins involved in defence (Leivar et al. 2011).

### Stress-related genes are upregulated in JA insensitive plants

At 72 hpi, two upregulated genes are detected in the JA insensitive plants, the first encoding sesquiterpene synthase 2 and the other annotated as encoding an ankyrin repeat-containing protein (Table 2). Both these genes are still upregulated at 120 hpi. In the JA insensitive plants, 37 genes are upregulated at 120 hpi, of which 12 are also up in the wild type and SA deficient plants (online resource 3). An additional 18 more genes are up in both JA insensitive and SA deficient plants. Ten genes are unique to the JA insensitive plants. Among the top upregulated genes at 120hpi in the JA insensitive plants is the previously mentioned sesquiterpene synthase gene, but also copalyl diphosphate synthase (Table 2). Both these genes are not unique to the JA insensitive plants and are also upregulated in the wild type and SA deficient plants. They encode enzymes involved in terpenoid biosynthesis (Degenhardt et al. 2009). Solanaceous plants are known to produce terpenoids as phytoalexins (Jadhav et al. 1991). Additionally, stress related genes encoding cytoplasmic small heat shock proteins from class I and AtHSFB3 (Arabidopsis thaliana heat shock transcription factor B3) were upregulated (Table 2). Among the ten DEGs unique to JA insensitive plants was an MLO1 homologue (PGSC0003DMG400020605), this same gene was found to be involved in stress responses and upregulated by ABA in potato (Wiesel et al. 2015).

### *Pathogenesis-related* genes are upregulated at 120 hpi in wild type and SA-deficient plants

After 120 hpi, we detected upregulation of *pathogenesis-related* genes in both the wild type and the SA-deficient, but not in the JA-insensitive plants (Table 2 and Online Resource 2). Among these are many genes encoding chitinases (PGSC0003DMG400010283; PGSC0003DMG400001528, PGSC0003DMG402001531), PR1 protein (PGSC0003DMG400005116), as well as Thaumatin (PGSC0003DMG400004259). Additionally, we also found two genes encoding glucan endo-1,3-beta-D-glucosidases (PGSC0003DMG400040260, PGSC0003DMG400029830), upregulated in the SA-deficient plants at 72 hpi. These two genes continue to be upregulated at 120 hpi and also appear upregulated in the wildtype at 120 hpi. Thus, upregulation of these *pathogenesis-related* genes during the infection of potato foliage with *A. solani* appear to not require intact SA signalling, but rather require JA signalling.

### SA deficient plants show many differentially expressed genes 120 hpi

At 120 hpi in the SA deficient line, we found the highest number of differentially expressed genes. Among the top 10 genes, included those encoding 4 PR1 proteins, Wound-induced protein WIN1, osmotin, lipoxygenase and glucan endo-1,3 beta-glucosidase, basic isoform 1 (Table 2). The upregulation of these genes is likely a reaction to the presence of a pathogen. Among the significantly enriched GO terms (Online resource 2) we find GO:0006952 ‘defence response’. In response to the *A. solani* infection, the SA deficient mutant also appears to try to compensate for the SA deficiency inflicted by the *NahG* transgene, since 3 upregulated genes (PGSC0003DMG400023458, PGSC0003DMG400019386, PGSC0003DMG401021564) encode a key enzyme in the SA biosynthesis pathway, Phenylalanine ammonia-lyase (PAL) (Chen et al. 2009). The upregulation of genes encoding PR-1 might be considered surprising*.* However, Halim et al. (2004) also found induction of a PR1a gene in *NahG* potato plants, even though no SA could be detected, indicating that this expression was SA-independent. Furthermore, in Arabidopsis many PR1 homologues are not SA regulated. Yu et al. (1997) showed that potato plants expressing the *NahG* gene do not show a significant difference in disease severity when infected with *P. infestans*, from which they hypothesise that potato has an inferior SA signal perception and/or transduction mechanism than both *A. thaliana* and tobacco. Additionally, Tsuda et al. (2013) showed that even though SA was required to induce PR1 expression in *A. thaliana* during PAMP (Pathogen Associated Molecular Pattern) Triggered Immunity (PTI), the regulation of SA was not required. The sustained activity of MAP kinases was, however, sufficient for the induction of PR1 expression during Effector Triggered Immunity (ETI) not only in the wild type but also in SA-induction deficient mutant *sid2.* Hence, the expression of PR-1 genes in the SA-deficient (*NahGD2)* plants could be due to the altered transduction mechanisms of SA signals in potato, or due to another pathway that is also capable of inducing expression of these genes. Another upregulated gene encodes potato kiwellin protein KiTH-2 (PGSC0003DMG400008101), this same gene was shown to be upregulated upon infection by *P. infestans* (Draffehn et al. 2013). Additionally, several studies showed that potato varieties with higher resistance to *P. infestans* showed increased expression of this gene (Draffehn et al. 2013; Ali et al. 2014; Mosquera et al. 2016). Han et al. (2019) recently showed that a maize homologue of KiTH-2 can block the active site of an effector secreted by the biotrophic fungus *Ustilago maydis*. They further suggest that kiwellins might be widespread proteins counteracting effectors secreted by plant pathogens. Interestingly, another study in our lab by Brus-Szkalej et al. (manuscript under review) found the same gene upregulated in potato plants (cv. Désirée) harbouring transgenic expression of the oomycete elicitor Pep13. Upregulation of KiTH-2 in SA-deficient *NahGD2* could potentially play a role in the increased susceptibility to *A. solani*, whereas it might be important for increased resistance against the hemibiotrophic pathogen *P. infestans.* Another potato gene that was previously shown to positively correlate with resistance to potato late blight is the hin1-like protein (StPOTHR1). It was found that the more resistant cultivars have higher expression of *StPOTHR1* and that overexpression of *STPOTHR1* results in increased resistance against *P. infestans* compared to the wild type (Chen et al. 2018). At 120 hpi in the SA-deficient plants we found two upregulated genes (PGSC0003DMG400028152 and PGSC0003DMG400028235) annotated as Hin1 proteins that show 99% and 96% protein identity to StPOTHR1 respectively. Chen et al. (2018) found that *StPOTHR1* transcripts were specifically induced at the inoculation site where the hypersensitive response (HR) occurs but were not essential for HR development. In a co-immunoprecipitation experiment in *N. benthamiana* the MAP kinase protein NbMKK5L was identified as an interactor of StPOTHR1, indicating that the increased resistance to *P. infestans* found in the *StPOTHR1* overexpressor plants could be associated with a MAP kinase signalling cascade.

### Wild type response to *A. solani* infection involves upregulation of transporters at 120 hpi

The uniquely upregulated genes in wild type potato were significantly enriched in GO terms with transport functions. One of these genes is annotated as copper transporter 1 (Table 3). Copper has long been known to have antimicrobial properties and has been applied in agriculture extensively since the discovery of the Bordeaux mixture in 1885 (Laminchhane et al. 2018). However, copper is also an essential micronutrient in plants. The copper sensitive bacterial pathogen of rice *Xanthomonas oryzae* pv *oryzae,* employs an effector to target a susceptibility factor, the XA13 protein, that interacts with two copper transporters (COPT1 and COPT5) to remove copper from the xylem vessels where the bacteria proliferate (Yuan et al. 2010). Other transporter genes that show upregulation are annotated as a zinc transporter, and an Iron-regulated transporter 1 (Table 3). Upregulation of transporter genes in response to *A. solani* could be part of a nutrient redistribution arising from an immune reaction of the plant.

### In wild type plants transferase encoding genes are downregulated

Among the downregulated genes are several transferase genes. Multiple UDP-glucuronosyltransferases, Serine-glyoxylate aminotransferase (SGA), Glucosyltransferase and a gene encoding cellulose synthase (PGSC0003DMG400011752) were downregulated (online resource 2). Inhibition of cellulose synthesis was shown to be required for alteration of the cell wall to promote pathogen defences in *A. thaliana* (Hernández-Blanco et al. 2007). The top downregulated genes encode oxidoreductases, belonging to the 2-oxoglutarate (2OG) and Fe(II)-dependent oxygenase family. Genes from this family are involved in the synthesis of multiple plant hormones such as gibberellic acid (Rieu et al. 2008; Schomburg et al. 2003), jasmonic acid (Caarls et al. 2017) and salicylic acid (Zhang et al. 2013, 2017). Interestingly, the extensively studied susceptibility gene *DMR6* also encodes a salicylic acid hydroxylase, belonging to the 2OG-Fe(II) oxygenase family and is involved in the fine-tuning of SA homeostasis (Zhang et al. 2017).

## Conclusions

In this study, we show for the first time that salicylic acid (SA) is involved in regulating symptom development in response to *A. solani* infection in both potato foliage as well as in tubers. Reducing symptom development in both the foliage and tubers requires intact SA signalling. Additionally, using a time course microarray analysis, we show that more genes are differentially regulated in the SA-deficient plants after *A. solani* infection compared to the wild type and JA insensitive plants. Only a small number of DEGs were found to overlap between the SA-deficient, JA-insensitive and wild type plants. In wild type plants, transporter genes were upregulated, whereas transferase genes were downregulated. Additionally, we found that pathogenesis-related genes are also upregulated when SA signaling is impaired, yet intact SA signalling in potato is required for defences against the necrotrophic pathogen *A. solani* since absence leads to larger lesions and higher fungal biomass.

## Electronic supplementary material

Below is the link to the electronic supplementary material.

Online resource 1 The larger *A. solani* lesion development in salicylic acid-deficient potato plant lines (*NahGD2* and *NahGA*) can be reversed by watering the soil with salicylic acid sodium salt (1 mM). Average lesion size (mm) at 10 dpi of two separate experiments (a) wild type (N = 10), *NahGD2* (N = 29), *NahGD2*+ SA (N = 28), *NahGA* (N = 35) and *NahGA* + SA (N = 32) and (b) *NahGD2* (N = 63), *NahGD2* + SA (N = 61), *NahGA* (N = 60) and *NahGA* + SA (N = 63). In both experiments the SA soil application significantly decreases the lesion size for both *NahG* lines compared to the control of the same line that was watered with tap water. Error bars represent the standard error of the mean. Letters represent the groups as determined by one-way ANOVA followed by Fisher’s Pairwise comparison’s test (p < 0.05). Plant lines that do not share a letter are significantly differentSupplementary file1 (PNG 122 kb)

Online resource 2 Complete table of all DEGs upon infection with *A. solani* at 24, 72, and 120 hpi in the wild type, the JA-insensitive (*coi1H1*), and SA-deficient (*NahGD2*) plant lines (XLSX 91 kb)

Online resource 3 Overlapping differentially expressed genes (DEGs) upon infection with *A. solani* at 120 hpi in the wild type, the JA-insensitive (*coi1H1*), and SA-deficient (*NahGD2*) plant lines (DOCX 33 kb)

Online resource 4 table with the Gene Ontology (GO) term enrichment analysis results obtained using AgriGO v2 (XLSX 56 kb)
