# Supplementary figures and images for: Intact salicylic acid signalling is required for potato defence against the necrotrophic fungus Alternaria solani
Source: Plant Mol Biol. 2020 Jun 19;104(1):1–19. doi: 10.1007/s11103-020-01019-6 (PMC7417411; doi:10.1007/s11103-020-01019-6)

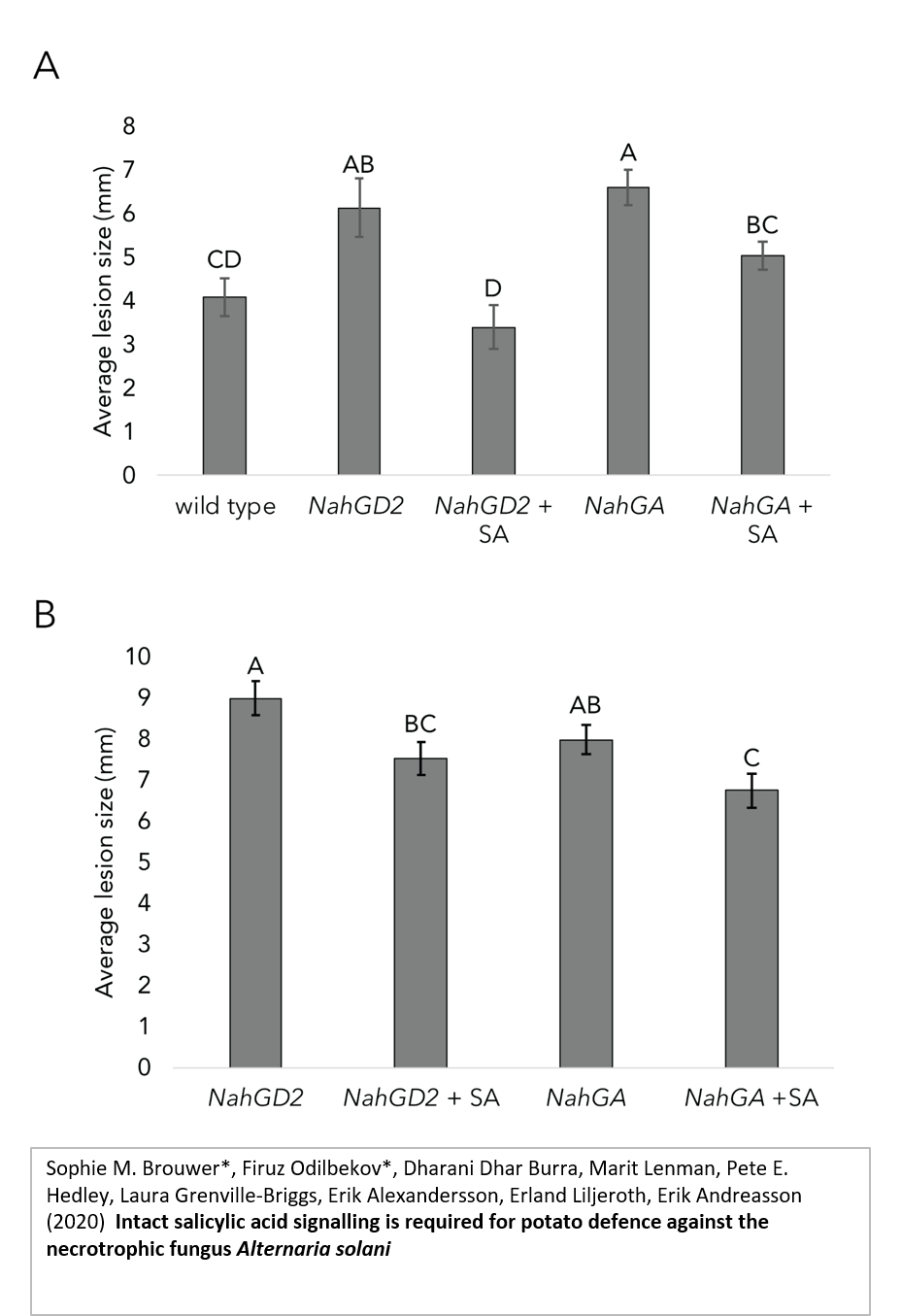

Supplement: Supplementary file 1 — Online resource 1 The larger A. solani lesion development in salicylic acid-deficient potato plant lines (NahGD2 and NahGA) can be reversed by watering the soil with salicylic acid sodium salt (1 mM). Average lesion size (mm) at 10 dpi of two separate experiments (a) wild type (N = 10), NahGD2 (N = 29), NahGD2+ SA (N = 28), NahGA (N = 35) and NahGA + SA (N = 32) and (b) NahGD2 (N = 63), NahGD2 + SA (N = 61), NahGA (N = 60) and NahGA + SA (N = 63). In both experiments the SA soil application significantly decreases the lesion size for both NahG lines compared to the control of the same line that was watered with tap water. Error bars represent the standard error of the mean. Letters represent the groups as determined by one-way ANOVA followed by Fisher’s Pairwise comparison’s test (p < 0.05). Plant lines that do not share a letter are significantly differentSupplementary file1 (PNG 122 kb) [file 11103_2020_1019_MOESM1_ESM.png]
