# Supplementary material for: Intact salicylic acid signalling is required for potato defence against the necrotrophic fungus Alternaria solani
Source: Plant Mol Biol. 2020 Jun 19;104(1):1–19. doi: 10.1007/s11103-020-01019-6 (PMC7417411; doi:10.1007/s11103-020-01019-6)
Supplement: Supplementary file 3 — Online resource 3 Overlapping differentially expressed genes (DEGs) upon infection with A. solani at 120 hpi in the wild type, the JA-insensitive (coi1H1), and SA-deficient (NahGD2) plant lines (DOCX 33 kb) [file 11103_2020_1019_MOESM3_ESM.docx]

| Gene Number (DMG) | Description | Log2 Fold Change | | |
| --- | --- | --- | --- | --- |
|  |  | **wild type** | **JA insensitive** | **SA deficient** |
| PGSC0003DMG400023922 | Cytoplasmic small heat shock protein class I | 4.47 | 3.08 | 3.77 |
| PGSC0003DMG400029201 | Sesquiterpene synthase 2 | 4.42 | 3.81 | 4.71 |
| PGSC0003DMG400001550 | TSI-1 protein | 3.74 | 2.70 | 5.07 |
| PGSC0003DMG400002027 | Cytoplasmic small heat shock protein class I | 3.61 | 2.86 | 3.44 |
| PGSC0003DMG400002028 | Cytoplasmic small heat shock protein class I | 3.60 | 2.88 | 3.39 |
| PGSC0003DMG400029830 | Glucan endo-1,3-beta-D-glucosidase | 3.30 | 2.23 | 3.77 |
| PGSC0003DMG400001948 | Copalyl diphosphate synthase | 3.24 | 2.58 | 4.24 |
| PGSC0003DMG400022929 | Aspartate aminotransferase | 2.85 | 2.73 | 3.53 |
| PGSC0003DMG400013763 | Ankyrin repeat-containing protein | 2.50 | 1.76 | 3.50 |
| PGSC0003DMG400018066 | Sesquiterpene synthase 2 | 2.46 | 1.57 | 2.77 |
| PGSC0003DMG400010490 | Acidic class II 1,3-beta-glucanase | 1.86 | 1.24 | 1.08 |
| PGSC0003DMG401010492 | Acidic class II 1,3-beta-glucanase | 1.45 | 1.34 | 2.25 |

Sophie M. Brouwer*, Firuz Odilbekov*, Dharani Dhar Burra, Marit Lenman, Pete E. Hedley, Laura Grenville-Briggs, Erik Alexandersson, Erland Liljeroth, Erik Andreasson (2020) **Intact salicylic acid signalling is required for potato defence against the necrotrophic fungus *Alternaria solani***

**Online resource 3** Overlapping differentially expressed genes (DEGs) upon infection with *A. solani* at 120 hpi in the wild type, the JA-insensitive (*coi1H1*), and SA-deficient (*NahGD2*) plant lines
